# Role of *Culex *and *Anopheles *mosquito species as potential vectors of rift valley fever virus in Sudan outbreak, 2007

**DOI:** 10.1186/1471-2334-10-65

**Published:** 2010-03-11

**Authors:** AlaaEddeen M Seufi, Fatma H Galal

**Affiliations:** 1 Department of Entomology, Faculty of Science, Cairo University, Giza, Egypt, 12211

## Abstract

**Background:**

Rift Valley fever (RVF) is an acute febrile arthropod-borne viral disease of man and animals caused by a member of the *Phlebovirus *genus, one of the five genera in the family *Bunyaviridae*. RVF virus (RVFV) is transmitted between animals and human by mosquitoes, particularly those belonging to the *Culex, Anopheles *and *Aedes *genera.

**Methods:**

Experiments were designed during RVF outbreak, 2007 in Sudan to provide an answer about many raised questions about the estimated role of vector in RVFV epidemiology. During this study, adult and immature mosquito species were collected from Khartoum and White Nile states, identified and species abundance was calculated. All samples were frozen individually for further virus detection. Total RNA was extracted from individual insects and RVF virus was detected from *Culex, Anopheles *and *Aedes *species using RT-PCR. In addition, data were collected about human cases up to November 24^th^, 2007 to asses the situation of the disease in affected states. Furthermore, a historical background of the RVF outbreaks was discussed in relation to global climatic anomalies and incriminated vector species.

**Results:**

A total of 978 mosquitoes, belonging to 3 genera and 7 species, were collected during Sudan outbreak, 2007. *Anopheles gambiae arabiensis *was the most frequent species (80.7%) in White Nile state. Meanwhile, *Cx. pipiens *complex was the most abundant species (91.2%) in Khartoum state. RT-PCR was used and successfully amplified 551 bp within the M segment of the tripartite negative-sense single stranded RNA genome of RVFV. The virus was detected in female, male and larval stages of *Culex *and *Anopheles *species. The most affected human age interval was 15-29 years old followed by ≥ 45 years old, 30-44 years old, and then 5-14 years old. Regarding to the profession, housewives followed by farmers, students, shepherd, workers and the free were more vulnerable to the infection. Furthermore, connection between human and entomological studies results in important human case-vulnerability relatedness findings.

**Conclusion:**

Model performance, integrated with epidemiologic and environmental surveillance systems should be assessed systematically for RVF and other mosquito-borne diseases using historical epidemiologic and satellite monitoring data. Case management related interventions; health education and vector control efforts are extremely effective in preparedness for viral hemorrhagic fever and other seasonal outbreaks.

## Background

Rift Valley fever (RVF) is an acute febrile arthropod-borne viral disease of man and animals caused by a member of the *Phlebovirus *genus, one of the five genera in the family *Bunyaviridae*. RVF virus (RVFV) is transmitted between animals and human by mosquitoes, particularly those belonging to the *Culex, Anopheles *and *Aedes *genera [1,2]. Transmission is mostly horizontal, but a vertical mode was described for some *Aedes *species [3,4]. RVFV is carried in the eggs of *Aedes *mosquitoes which breed in isolated depressions called *dambos *found in the vast grassland areas. At flooding of the *dambos *during periods of extensive and widespread rainfall, the eggs of the *Aedes *mosquitoes hatch and the subsequent adults transmit the virus to domestic animals including sheep, goats, cattle, camels, and buffalos [5]. These depressions also serve as good habitats for *Culex *and *Anopheles *mosquito species. When *Aedes *mosquitoes infect domestic animals with RVFV, virus amplification occurs in these vertebrate hosts, leading to propagation into various *Culex *and *Anopheles *species that are capable of transmitting the virus to a wider area beyond the area of the original outbreaks by wind-borne dispersal [6]. Although RVFV is, in most cases, transmitted to humans by mosquitoes, it may also be transmitted through direct contact with secretions of infected animals and meat. The professional nature of some groups, such as doctors and veterinarians working in slaughterhouses, made them more vulnerable than others to infection (because the virus is not vital outside the body). Good cooking of meat may help to eliminate the virus from it [7].

The disease in animals is characterized by high rates of abortion and death of young ruminants [1]. Human disease symptoms are often limited to a flu-like syndrome with transient fever, rigor (shivering), headache, severe muscle and joint pains, photophobia and anorexia, sometimes with a petaechial rash, nausea, vomiting and epistaxis. The symptomatic course is 4 to 7 days leading to full recovery in 2 weeks. However in severe cases, case fatality rates are about 1% and usually associated with hemorrhagic manifestations of the disease. Meningoencephalitis also occurs in about 1% of infections, but does not usually result in death. Ocular sequellae occur in a moderate percentage of cases and may result in impaired vision or even blindness, which might be permanent [2]. Human cases are mainly caused by direct contact with infected animal body fluids after abortion or slaughtering viremic animals and by mosquito bites. When an epizootic occurs in animals, it is easily transmitted to humans leading to an epidemic [8].

RVFV was first identified in 1931 during an investigation into an epizootic among sheep on a farm in the Rift Valley of Kenya. Since then, outbreaks have been reported in sub-Saharan and North Africa. Data presented in Table 1 described all **previously reported RVFV outbreaks **after 1931. In 1950-51, a major epizootic in Kenya caused 500,000 abortion and 100,000 deaths in sheep. In 1977, an epidemic of RVF was recoded in Egypt. *Cx. pipiens *was incriminated in disease transmission and dissemination [9,10]. A major outbreak occurred in Senegal in 1987 and 2003. Both *Cx. poicilipes *and *Ae. vexans *were collected from the affected sites [11]. In 1997-98, Faye *et al*. reported a major outbreak occurred in Eastern Africa (*Cx. theileri*), Kenya (*Cx. zombaensis, An. coustani, Ae. mcintoshi, M. africana, and M. uniformis*), Somalia, Mauritania (*An. pharoensis, An. rhodesiensis, An. rufipes, Cx. antennatus, Cx. decens, Cx. neavei, Cx. perfuscus, Cx. poicilipes, Cx. quinquefasciatus*, Sandflies, Biting midges) and Tanzania (*Ae. mcintoshi*) [11]. Mauritania registered another outbreak in 2003. *Cx. poicilipes, Cx. antennatus*, and *M. uniformis *were incriminated in disease transmission [11]. Additional major outbreak was recorded in Kenya, Tanzania, Sudan and Somalia [8,12]. In September 2000, RVF cases were confirmed in Saudi Arabia (*Cx. pipiens *complex, *Ae. vexans arabiensis, Ae. Vittatus, Ae. (Stegomyia) nilineatus *and *Cx. (cx.) triteniorynchus*) and Yemen, marking the first reported occurrence of the disease outside the African continent and raising concerns that it could extend to other parts of Asia and Europe [13-15]. During these multiple outbreaks and epidemics, the virus exhibited an amazing flexibility to adapt to different ecological contexts and to take advantage of climatic change and environmental disruptions (dam building, land irrigation, etc.). Such an adaptive ability resulted in a high toll of deaths, ailments and economic losses during its recent emergence episodes [16].

**Table 1:** Historical background on the recorded outbreaks in Africa and Asia continents showing the year of outbreak, country and incriminated arthropod species.

Year of outbreak	Affected country	Collected arthropods	Reference
1997-98 and December 2006	Kenya	Culex zombaensis, Anopheles coustani, Aedes mcintoshi*, Mansonia africana, and M. uniformis	[8]

1997-98 and from January to May 2007	Tanzania	Aedes mcintoshi*	[12]

1997-1998	Eastern Africa,	Culex theileri*	[11]

1977	Egypt	Culex pipiens*	[9,10]

1987, 2003	Senegal	Culex poicilipes* and *Aedes vexans*	[11]

23 Oct 2000	Kingdom Saudi Arabia	*Culex pipiens *complex, *Aedes vexans arabiensis, Ae. Vittatus, Ae. (Stegomyia) nilineatus, Aedes vexans arabiensis and Culex (culex)* triteniorynchus*	[13,14,16]

19 Oct 2000	Yemen	Not defined	[15]

1987, 1998 and October 2003	Mauritania	An. pharoensis, rhodesiensis, rufipes, C. antennatus, decens, neavei, perfuscus, poicilipes, quinquefasciatus*, Sandflies, Biting midges. Culex poicilipes*, antennatus, Mansonia uniformis (2003)	[11,34,35]

Oct, 2007 to Jan, 2008	Sudan.	*Culex pipiens *complex, Anopheles gambae arabiensis*, Ae. aegypti.	[8,36]

1997-98 and Dec 2006 to Feb 2007	Somalia	Not defined	[12]

*Most abundant species in the collection.

Since livestock immunization against RVF has so far appeared difficult to implement efficiently in areas of endemicity, strengthened surveillance, early detection, management of cases seemed to be among the best options to prevent extension of RVF epidemic foci. Precise estimation of specific weight for each risk factor is a considerable guide to construct an effective outbreak control plan. Insufficient entomological studies and surveys were conducted and resulted in a vague evaluation of the vector role in RVFV transmission and dissemination in Sudan. For that reasons, this study was conducted to answer some raised questions about the role of vector(s) in RVFV outbreak, 2007 in Sudan.

## Methods

### Insect collection and identification

Adult and immature stages of mosquitoes were collected on December 2007 from their breeding sites in White Nile (affected) and Khartoum (not affected) states. Adults were collected using CO_2_-baited light traps and aspirators while immature stages were collected by sieving potential larval habitats with dippers. 476 and 502 mosquitoes were collected from White Nile and Khartoum states, respectively. All specimens were identified at Sudan's National Health Laboratory. Mosquitoes were pooled concerning their site of collection, species, sex and stage (larva, pupa or adult). Mosquitoes were then frozen for further virus detection. In both collection sites, livestock (sheep, goats, cattle, camels and cows) were housed at night in very close contact to their owners.

### Molecular detection of RVFV in mosquitoes

#### RNA extraction

Total RNA was extracted from individual insects using RNeasy kit according to manufacturer's instructions (QIAGEN). The RNA was dissolved in DEPC-treated water, quantified spectrophotometrically and analyzed on 1.2% agarose gel.

#### Reverse transcription of RNA

For synthesis of first strand cDNA, reverse transcription reactions were performed using oligo dT primer (5'-TTTTTTTTTTTTTTT-3'). Each 25 μl reaction mixture containing 2.5 μl of 5× buffer with MgCl_2_, 2.5 μl of 2.5 mM dNTPs, 1 μg of primer, 2 μg RNA, 200 U reverse transcriptase enzyme. RT-PCR amplification was performed in a thermal cycler (Eppendorf) programmed at 42°C for 1 hr, 72°C for 10 min. cDNA was then stored at -20°C until used.

#### PCR amplification using RVFV specific primer set

Total PCR reaction volume of 25 μl containing 10 mM Tris HCl (pH 8.3), 25 mM KCI, 4 mM MgCl_2_, 200 μM dNTPs, 1 U *Taq *DNA polymerase (AmpliTaq, Perkin-Elmer), 2 μl of the 10 pmol primer and 1 μl of the product of reverse transcriptase reaction was employed. PCR primers for this assay (Fwd: 5'-GAC TAC CAG TCA GCT CAT TAC C-3' and Rev: 5'-TG TGA ACA ATA GGC ATT GG-3') were designed to anneal to G_2 _glycoprotein region within the M segment of the tripartite negative-sense single stranded RNA genome of RVFV. PCR reaction was cycled first in a 9700 thermal cycler (Perkin-Elmer) programmed at 94°C for 5 min (one cycle), then followed by 40 cycles at 94°C for 15 s, 50°C for 15 s, and 72°C for 30 s. Reaction was then incubated at 72°C for 10 min for final extension [17]. Two μl of loading dye were added prior to loading of 10 μl per gel pocket. Electrophoresis was performed at 80 Volt with 0.5 × TBE buffer in 1.5% agarose gel. Gel was stained in 0.5 μg/ml (w/v) ethidium bromide solution. Finally, gel was visualized and photographed by using gel documentation system.

### Human studies

#### Case definitions

A suspected human RVFV case-patient was defined as a person with fever associated or not with hemorrhagic jaundice, neurological symptoms or any person who died with overt hemorrhagic fever symptoms from October through December 2007. A confirmed human RVFV case-patient was defined by more than one of these laboratory tests: immunoglobulin M (IgM), RT-PCR or virus isolation positive results.

#### Human investigations

In affected areas, the investigation was conducted under the supervision of the chief of the sanitary district. For each case, blood samples were collected and an interview in which information was gathered about sex, age, date of fever onset, profession and hemorrhagic symptoms for all case-patients and their contacts. All data of human studies were kindly supported by the Sudan's National Health Laboratory, Department of Epidemiology at the Federal Ministry of Health, Sudan government.

#### Historical background on RVFV outbreaks

Outbreaks of RVFV were followed up through publications and reports. Table 1 presented a complete history of the reported epizootics of RVFV, including the year of outbreak, affected countries, collected arthropods that were incriminated in disease transmission and dissemination and the reference reporting the epizootics.

## Results

### Entomological results

A total of 978 mosquitoes, belonging to 3 genera and 7 species, were collected (Tables 2 and 3). Among the species collected from White Nile state, *Anopheles gambiae arabiensis *was the most frequent species (80.7%), followed by *Culex pipiens *complex (8.8%), *Aedes aegypti *(5.0%), *An. coustani *(2.9%), *Cx. poicilipes *(1.7%), then both *Ae. mcintoshi *and *Ae. vexans *(0.4%). Meanwhile, *Cx. pipiens *complex was the most abundant species (91.2%) collected from Khartoum state, followed by *Cx. poicilipes *(4.4%), *An. gambiae arabiensis *(3.2%), *An. coustani *(0.8%) and *Ae. mcintoshi *(0.4%). Neither *Aedes aegypti *nor *Ae. vexans *was collected from Khartoum. Overall, *Cx. pipiens *complex was more abundant than *An. gambiae arabiensis*. Immature and adult species collected from the two sites are presented in Tables 2 and 3. A total of 26 monospecific pools were constituted regarding to species, stage and site of collection. Individual mosquitoes were then submitted to RT-PCR assay for RVFV detection.

**Table 2:** A key table indicates the collected species number, stage, abundance and positive RT-PCR samples from White Nile state.

Species	Total no. of collected species	Stage				Species abundance*	No. of positive PCR samples
				
		Larva	Pupa	Male	Female		
*An. gambiae arabiensis*	384	28	4	30	322	80.7%	3L+2 M+26F** = 31

*An. coustani*	14	2	-	2	10	2.9%	1L+2F = 3

*Cx. pipiens *complex	42	22	4	-	16	8.8%	4L+1F*** = 5

*Cx. poicilipes*	8	6	-	-	2	1.7%	1F = 1

*Ae. aegypti*	24	22	-	-	2	5.0%	8L+2F**** = 10

*Ae. mcintoshi*	2	-	-	-	2	0.4%	0

*Ae. vexans*	2	2	-	-	-	0.4%	0

Total	476	82	8	32	354	100%	50

* Abundance = number of individuals of one species/total number of mosquitoes collected (approximated to one decimal).

** 24 gravid + 2 ungravid females.

*** Gravid females.

**** Ungravid female.

**Table 3:** A key table indicates the collected species number, stage, abundance and positive RT-PCR samples from Khartoum state.

Species	Total no. of collected species	Stage				Species abundance*	No. of positive PCR samples
				
		Larva	Pupa	Male	Female		
*An. gambiae arabiensis*	16	16	-	-	-	3.2%	2L = 2

*An. coustani*	4	2	-	-	2	0.8%	-

*Cx. pipiens *complex	458	446	-	4	8	91.2%	22L+2F** = 24

*Cx. poicilipes*	22	16	2	-	4	4.4%	-

*Ae. aegypti*	-	-	-	-	-	0.0	-

*Ae. mcintoshi*	2	2	-	-	-	0.4%	-

*Ae. vexans*	-	-	-	-	-	0.0	

Total	502	482	2	4	14	100%	26

* Abundance = number of individuals of one species/total number of mosquitoes collected (approximated to one decimal).

** Ungravid female.

### RT-PCR amplification using RVFV specific primer set

**Figure 1:**
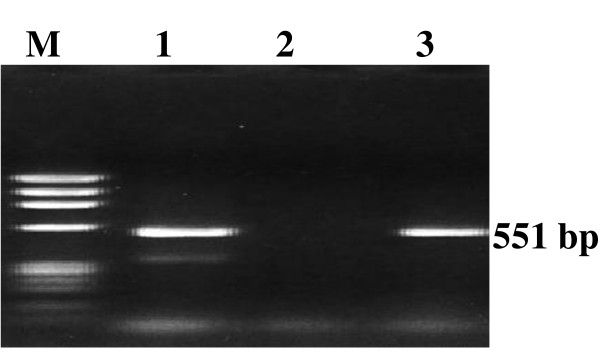
**1·5% agarose gel electrophoresis showing RT-PCR product (551 bp) of RVFV in infected mosquitoes**. Lanes M, 1, 2 and 3 show *PhiX174 HaeIII *DNA marker, 551 bp amplified product from RVFV-infected mosquito, PCR mix without DNA (as negative control) and ELISA positive mosquito (as positive control), respectively.

Two oligonucleotide primers were designed to amplify 551 bp within the M segment of the tripartite negative-sense single stranded RNA genome of RVFV [17], and were successfully used in RT-PCR. Electrophoretic analysis of the RT-PCR product on agarose gels revealed that the primers amplified a single band of expected size of 551 bp only in the RNA extracted from RVF-infected samples. PCR mix with no template was used as negative control (Fig. 1). RVFV was successfully detected in larvae and females of *An. gambiae arabiensis, An. coustani, Cx. pipiens *complex and *Ae. aegypti *collected from White Nile state (Table 2). Infection rate was estimated as (31/476) 6.5, (3/476) 0.6, (5/476) 1.1 and (10/476) 2.1%, respectively. No virus was detected in either *Ae. mcintoshi *or *Ae. vexans*, while only one female of *Cx. poicilipes *was tested positive for RVFV collected from White Nile state (Table 2). In parallel, RVFV was successfully detected in both larvae and females of *Cx. pipiens *complex and *Cx. poicilipes *collected from Khartoum state (Table 3). Infection rate was estimated as (20/502) 4 and (4/502) 0.8%, respectively. No virus was detected in *Ae. mcintoshi *collected from Khartoum state. Two larvae, one of *An. gambiae arabiensis *and one of *An. coustani *were tested positive for RVFV collected from Khartoum state (Table 3). It is noteworthy to mention that only two male samples of *An. gambiae arabiensis *were tested positive for RVFV collected from White Nile state (Table 2).

### Human results

The different cases recorded in the affected states (White Nile, Gazira and Sinnar) from the first onset up to Nov 24^th^, 2007 are presented in Table 4. Gazira state hospitals received the highest number of suspected cases (287/451), followed by White Nile (111/451) and Sinnar (53/451) states. The cumulative number of deaths was arranged in the same order. The first cases appeared in southern areas of Algabalain locality in White Nile state at October 4^th^, 2007. According to the doctors and citizens in that area, the first symptoms were hemorrhage and fever with rapid death. All reported cases in the beginning of the outbreak were scattered and did not reach any health facilities. After that the cases started to be reported from Sinnar then Gazira states. It was observed that the number of infections and subsequent deaths were more frequent in males than females (Table 5). The most affected age interval was 15-29 years old followed by ≥ 45 years old, 30-44 years old, and then 5-14 years old. Regarding to the profession, housewives followed by farmers, students, shepherd, workers and the free were more vulnerable to infection than others (Table 5). Majority of cases appeared with hemorrhagic symptoms. 2-5% of all cases exhibited eye complications and 1% exhibited meningococcal meningitis. From the 451 reported case patients, 74 blood samples were submitted to ELISA and 5 samples to real time PCR investigation. Only 30 samples of ELISA and one sample of PCR were tested positive (Table 4). The overall case-fatality rate (CFR) was estimated as 42.8% in regard to the 451 reported cases (Table 4). Regarding to the fact that the case-fatality rate for infections with RVFV in humans is about 1%, the expected population infected with RVFV is 19303 cases.

**Table 4:** Epidemiological situation of human cases recorded in the affected states up to Nov 24^th^, 2007.

State	Cumulative			Laboratory results		
	
	Cases	Deaths	CFR%*	Total samples	Positive cases	
					
					ELISA	PCR
White Nile	111	65	58.6	27+2	11/27	0/2

Gazira	287	113	39.4	34+3	15/34	1/3

Sinnar	53	15	28.3	13	4/13	-

Total	451	193	42.8	74+5	30/74	1/5

*CFR: Case Fatality Rate (approximated to one decimal).

Cumulative cases, deaths, case fatality rate in relation to laboratory results are shown.

**Table 5:** Relationship between RVFV infection and some observed parameters among 451 cases.

Parameter	<5 years	5-14 y	15-29 y	30-44 y	> 45 years	
**Age**	2	32	159	120	138	

**Gender**	290 ♂			161 ♀		

Profession*	128 HW	100 F	66 S	38 Sh	21 Free	73 Others

*HW: housewives, F: Farmer, S: Student and Sh: Shepherd.

## Discussion and Conclusions

This work was developed by an expert Egyptian team on request of the Sudanese Federal Ministry of Health and the author (A.S.) was one of this team.

The low diversity of the mosquito species collected in this survey may be due to natural abundance of mosquito species (high abundance of *Culex *and *Anopheles *species). Alternatively, it might be because the mosquito captures were undertaken at the end of the rainy season, when most breeding sites had dried up and/or due to the intensive use of pesticides to control blood sucking insect vectors during outbreak. This hypothesis was further strengthened by the small number of mosquitoes caught (978) and the low number of *Aedes *mosquitoes, a vector species of RVFV in West Africa [18]. Among all adult and immature mosquito stages collected, *Cx. pipiens *complex (51.1%) and *An. gambiae arabiensis *(40.9%) were the most represented species. Most of the collected mosquito species, especially *Culex *species [19], were previously incriminated in RVFV transmission and dissemination (Tables 1, 2 and 3). In addition, feeding preference studies showed that *Cx. pipiens *fed preferentially on pigeons but also on human at indoors. However, *Anopheles *species fed preferentially on human but also on other mammals. These considerations on *Culex *and *Anopheles *biology and ecology as well as its experimentally demonstrated vectorial competence to RVFV (Table 1) constituted clues pointing to their relative role in RVFV transmission cycles. Excluding gravid females (to avoid false positive results), the relative role of *An. gambiae arabiensis *in transmission of RVFV in White Nile and Khartoum states was 1.5% and 0.4%, respectively. Meanwhile, the relative role of *Cx. pipiens *complex in transmission of RVFV was 0.8% and 4.8% in White Nile and Khartoum states, respectively. In case of White Nile state, the relative roles of *An. coustani*, *Cx. poicilipes *and *Ae. aegypti *were 0.6, 0.2 and 2.1%, respectively. High relative role in case of *Ae. aegypti *may be attributed to the high number of positive detections in relation to the number of collected samples. Although the choice of a 1-month period, for monitoring mosquito natural abundance is quite a short time, it was assumed to be a critical period for RVFV outbreak. In addition, we were quite a bit late in the transmission season and tried to follow up the outbreak as soon as we could launch it.

RT-PCR assay is considered as specific, sensitive tool for RVF diagnosis in the early phase of the disease and its results do not differ significantly from those obtained by virus isolation [20]. Successful RT-PCR detection of RVFV in female mosquito species was considered as a precursor for viral circulation in these species (incriminated in dissemination or acquired the virus in its midgut only). Meanwhile, successful RT-PCR detection of RVFV in male and larval stages, although at very low rates, referred to transovarial (vertical) transmission of the virus within these mosquito species. It may also refer to possible venereal RVFV transmission when a male being infected vertically and then infecting the female during mating. RVFV was known to be carried in the eggs of *Aedes *mosquitoes which can survive for several years in the dried mud [21]. These mosquitoes breed in isolated depressions called *dambos *found in the vast grassland areas. On flooding, *Culex *mosquito species (bread in *dambos*) play important role in virus circulation [22,23]. The survival of RVFV during interepizootics was believed to depend on transovarial transmission of the virus in floodwater *Aedes *mosquitoes [4].

Satellite monitoring (June-September, 2007) showed that most of the central Sudan could be unusually subjected to heavy rainfall, and generated RVF risk warnings for central and southern Sudan in July-September. In late October, RVF outbreaks were reported by World Health Organization (WHO) in humans in Sudan in White Nile, Sinnar, and Gazira states. By early November 2007, 329 human cases, including 96 deaths were reported. The cases being reported in Gazira State are in an area close to irrigation canals and are linked to naturally occurring cycles involving livestock and mosquitoes which are abundant in the irrigation zone. In parallel, many previously studied outbreaks were reported to occur during heavy rainfall seasons in various sub-Saharan countries including Kenya, Somalia, Tanzania, Senegal, Eastern Africa, Mauritania, Egypt and more recently in Saudi Arabia and Yemen. Earlier works suggested that epizootics and epidemics of RVFV occur periodically after heavy rains that flood natural depressions in the grasslands of sub-Saharan Africa [24,25]. In addition to three outbreaks recorded in neighboring countries (Table 1), Sudan was subjected to the heaviest rainfall season reported in the last forty years. Given the wide geographic and ecological range of RVFV, it is necessary to monitor large areas for conditions that may trigger the emergence of mosquito vectors that could spread RVF. Furthermore, there has been increased scientific interest in the connection between global climatic anomalies and disease outbreaks [26]. RVFV is one example of a disease whose outbreaks have been shown to be closely coupled with climate anomalies [25].

Overall connection between human and entomological results with regard to recorded outbreaks in neighboring countries were definite warnings for the incoming RFV outbreak in Sudan, especially that conditions of emerging epidemics were almost certain. Furthermore, the potential of RVF as a disease emerging in new areas was first documented in Egypt in 1977 [2], and since then, epidemics have occurred in Mauritania (1987 to 1988 and 1998), Madagascar (1990 to 1991), Egypt (1993), and eastern Africa (in Kenya, Somalia, and Tanzania) [27,28]. Recently, the outbreak on the Arabian Peninsula (in Yemen and Saudi Arabia) represented the first case of RVF outside Africa [29,30].

Although virus isolation (VI) is considered as gold standard method, IgM-ELISA method avoids false positive results due to the presence of rheumatoid factor and antinuclear antibodies. On the other hand, anti-RVF IgM antibodies were estimated to persist at a detectable level for up to 6 months in chronic infections [31]. However, combination of ELISA and RT-PCR assays is very important for rapid and efficient identification of RVFV during outbreaks. The present data and those obtained during the epidemics of RVF in Mauritania [11], in Kenya [32], as well as in Saudi Arabia and Yemen [33] demonstrated the importance of combining diagnostic assays for accurate and comprehensive detection of RVFV infection. The present results indicated that males of 15-29 years old were more susceptible than females. In parallel, housewives and farmers were the most susceptible people to RVFV infection (Table 5). These results may be related to their more vulnerability to the vector as well as to socioeconomic/professional activities which allow a direct contact with infected animals. Agreeable results were presented by Woods *et al*. [32] who stated that children <15 years of age were significantly less likely to have had recent RVFV infection. A relation between virus infection and direct contact with animals was reported by Faye *et al*. [11]. Indeed, those at highest risk include butchers and others who come in contact with animals (e.g., slaughterhouse workers, tanners, and herdsmen), who represent a large part of the population living in these areas. Although WHO estimated that the human mortality rate due to RVFV is ≈1%-2% of infected patients, the number of recorded deaths during this outbreak was 193 among 451 infected patients when the laboratory data were considered exclusively. This means that the number of infected but not officially recorded is about 19303 cases.

Conclusively, the present work reported that *Culex *and *Anopheles *were collected in relatively high numbers during Sudan outbreak, 2007. Because the mere presence of a mosquito species during the outbreak does not mean that it is involved in transmission cycle of RVFV, RT-PCR was used and successfully detected RVFV in female, male and larval stages of *Culex *and *Anopheles *species. Furthermore, connection between human and entomological studies results in important human case-vulnerability relatedness findings. It is noteworthy that validated RVF forecast models may provide early warning (~3 months) for RVF epidemics in Africa. Model performance, integrated with epidemiologic and environmental surveillance systems, should be assessed systematically for RVF and other mosquito-borne diseases using historical epidemiologic and satellite monitoring data. Case management related interventions; health education and vector control efforts are extremely effective in preparedness for viral haemorrhagic fever and other seasonal outbreaks. Further confirmatory transmission studies are very important to determine the vectorial capacity of the incriminated species in this study.

## Competing interests

The authors declare that they have no competing interests.

## Authors' contributions

AMS: Designed the overall study, carried out the molecular genetic studies optimization, performed data analysis, interpretation and manipulation, drafted and revised the manuscript, conceived of the study and participated in its coordination. FHG: Participated in the design of the study, participated in the optimization of molecular genetic studies, performed data analysis and interpretation, participated in editing of the manuscript, conceived of the study and participated in its coordination. All authors have read and approved the final manuscript.

## Pre-publication history

The pre-publication history for this paper can be accessed here:

http://www.biomedcentral.com/1471-2334/10/65/prepub
